# In vivo adaptive optics retinal imaging reveals rod-mediated cone photoreceptor disorganization in *RHO*-associated retinitis pigmentosa

**DOI:** 10.1038/s44385-026-00108-3

**Published:** 2026-09-02

**Authors:** Annette Kaminaka, Tao Liu, John P. Giannini, Natalie Toth, Diego Dominguez, Nancy Aguilera, Furu Zhang, Joanne Li, Bin Guan, Brett G. Jeffrey, Alfredo Dubra, Wadih M. Zein, Laryssa A. Huryn, Johnny Tam

**Affiliations:** 1 National Eye Institute, National Institutes of Health, Bethesda, MD USA; 2 Department of Ophthalmology, Stanford University, Palo Alto, CA USA

**Keywords:** Diseases, Medical research, Neuroscience

## Abstract

Pathogenic variants in the rhodopsin (*RHO*) gene are the most common cause of autosomal dominant retinitis pigmentosa leading to photoreceptor degeneration. Quantitative imaging using adaptive optics (AO) revealed irregularities in cone organization, even in relatively well-preserved retinal locations. At the leading disease front, rod density was disrupted to a greater extent than cones. Repeated longitudinal measurements demonstrate the possibility of using cone-based metrics for treatment trials to preserve photoreceptor structure.

*RHO*-associated retinitis pigmentosa (*RHO*-RP) is the most common cause of autosomal dominant retinitis pigmentosa^1,2^, driven by pathogenic mutations in rhodopsin (*RHO* gene, MIM *180380), a light-sensitive G-protein-coupled receptor within rod photoreceptors and a vital component of the visual transduction pathway^2–4^. As a consequence of rod degeneration, cone photoreceptors also degenerate with disease progression, due in part to cone dependence on rod trophic factors, prolonged cone starvation, and/or oxidative stress^4,5^. Recent innovations in adaptive optics (AO) retinal imaging^6,7^ have enabled the noninvasive, in vivo assessment of the human retina with cellular-scale resolution. AO imaging studies investigating various forms of retinitis pigmentosa have shown reduced cone density^8–10^, disrupted cone mosaic regularity^11^, and variable cone enlargement with escalating disorganization near the transition zone^9,12^. We hypothesized that direct visualization of both cone and rod photoreceptors in the living human eye alongside conventional retinal imaging could be useful for revealing the extent to which rods and cones are disrupted in situ across the retina (Fig. 1), considering both the leading disease front as well as clinically well-preserved retina areas in patients with sectoral *RHO-*RP (sRP) (Table 1 and Supplementary Fig. 1), a subset of *RHO*-RP in which the clinical characteristics of *RHO-*RP are initially observed in only one or two quadrants or sectors of the retina (typically inferior or inferonasal)^13^.

**Fig. 1:**
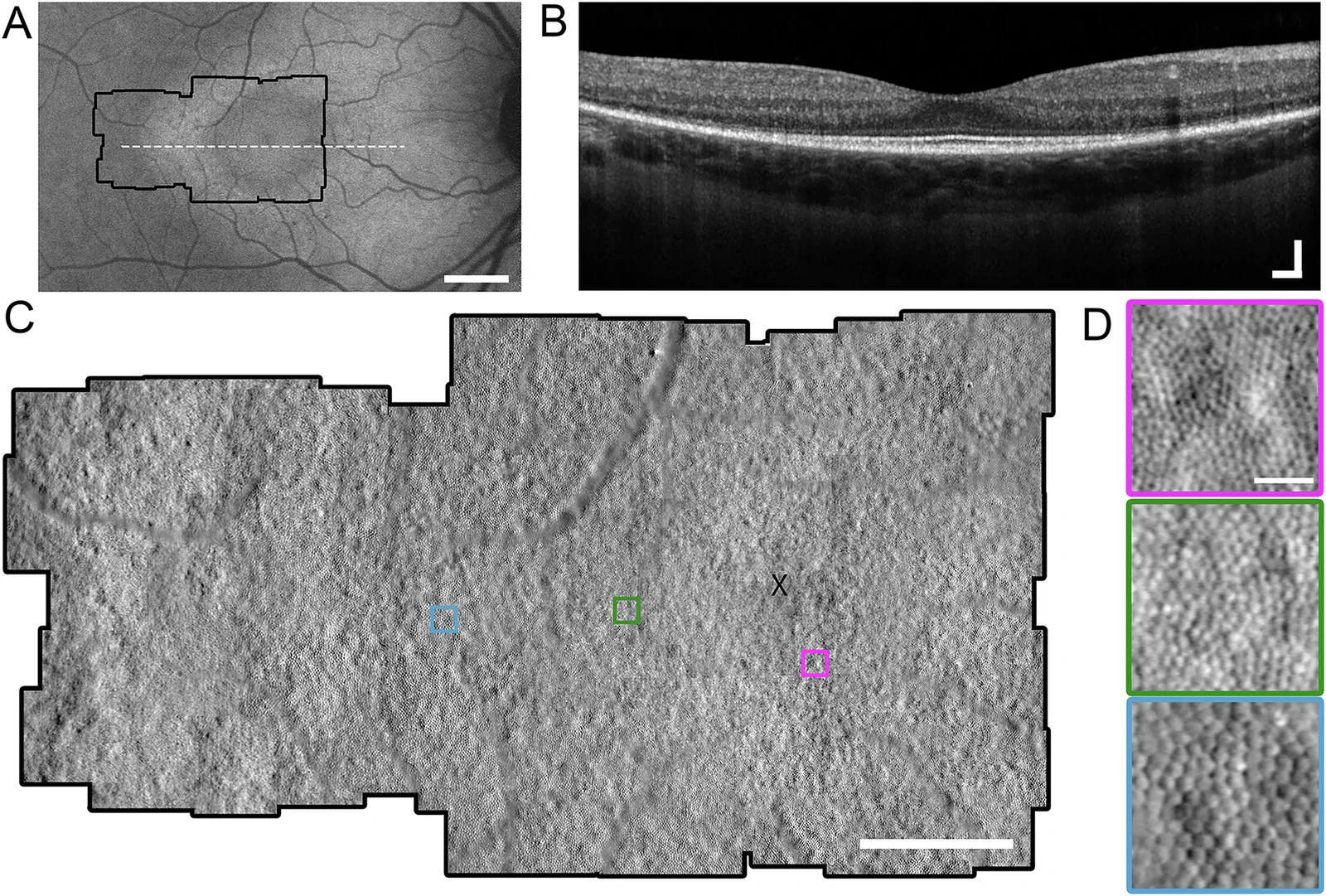
Multimodal imaging of *RHO*-RP with co-registration of non-confocal split detection adaptive optics (AO) images. **A** Near infrared autofluorescence (NIRAF) image of S7R (right eye of participant S7). Scale bar, 1 mm. **B** Spectral domain optical coherence tomography (SDOCT) B-scan through the dotted white line in (**A**) showing intact outer retinal photoreceptor bands throughout the preserved retinal regions. Horizontal and vertical scale bars, 200 µm. **C** Montage constructed of 76 overlapping split detection AO images of the area outlined in black in (**A**). The small black “X” denotes the fovea. Scale bar, 500 µm. **D** Zooms of AO images corresponding to the colored boxes in (C) at retinal eccentricities of 0.3 mm (magenta), 0.6 mm (green), and 1.2 mm (blue).

**Table 1:** Subject information

ID	Family	Sex	No. of visits	Age at first visit	Pathogenic *RHO* variant^a^	Class	sRP	BCVA (OD,OS)^b^	Axial length (mm; OD,OS)
S1	F1	F	3	46	c.50 C > T p.(Thr17Met)	2/4	Y	20/16, *20/20*	23.41, 23.36
S2	F1	F	3	48	c.50 C > T p.(Thr17Met)	2/4	Y	20/16, 20/20	24.07, 23.93
S3	F2	F	1	28	c.316 G > A p.(Gly106Arg)	2/2	Y	20/16, *20/16*	23.01, 22.89
S4	F3	F	3	57	c.68 C > A p.(Pro23His)	2	Y	20/20, 20/12	25.01, 25.17
S5	F4	M	3	54	c.44 A > G p.(Asn15Ser)	2	Y	20/20, *20/25*	25.44, 24.55
S6	F5	M	3	54	c.647 T > A p.(Met216Lys)	2?	N	20/25, 20/16	24.43, 24.39
S7	F6	F	1	21	c.1039 C > G p.(Pro329Ala)	N/A	N	20/16, 20/16	24.68, 24.44
S8	F6	M	1	26	c.1039 C > G p.(Pro329Ala)	N/A	N	20/16, *20/16*	23.93, 23.89

^a^All heterozygous. RHO transcript: NM_000539.3.

^b^Gray italics indicates that this eye was not assessed using AO.

## Adaptive optics imaging reveals changes in cone structure across preserved retinal areas

**Fig. 2:**
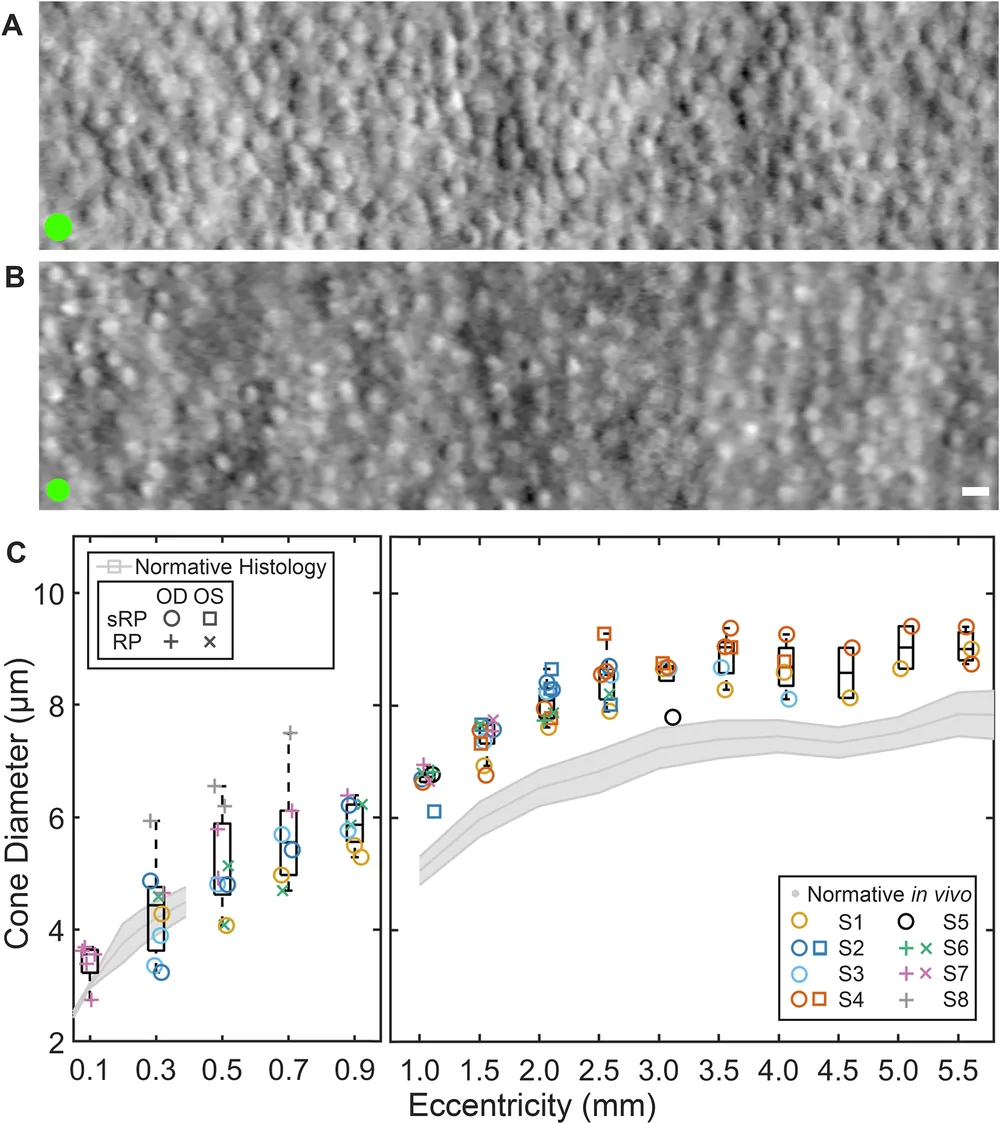
Cone inner segment diameters are enlarged in *RHO-*RP. **A** Example non-confocal split detection adaptive optics (AO) image of cone photoreceptor inner segments (3.0 mm eccentricity from S1R). **B** Example image from an age-, sex-, and axial length-matched healthy right eye (both from 3.0 mm eccentricity). Scale bar for both (**A**) and (**B**), 10 µm. The slightly enlarged cones in the *RHO*-RP image are irregularly clustered with variable spacing between neighboring cones. Green circles indicate the approximate size of a cone within each of the two images. **C** Cone diameter measurements from the *RHO-*RP cohort (each colored symbol is the average of three or more cone diameter ROIs measured at each eccentricity for one eye; see inset legend) in comparison to normative data. Histological data^7^ is not available between 0.4 and 0.9 mm and normative in vivo data^14,15^ is only available starting at 1.0 mm (gray shaded areas indicate 95% confidence intervals (CI) around the mean).

Noninvasive AO imaging revealed significant enlargement of cone photoreceptor inner segments in *RHO*-RP despite an intact, contiguous cone mosaic, even in preserved retinal areas (Fig. 2). Cones were least enlarged at the fovea (area with the fewest rods; eccentricity ≤0.5 mm), except in participant S7 (Fig. 1), who had visibly enlarged cones near the fovea. On average, cones were enlarged by 18.6 ± 6.5% compared to normal^14,15^ (*P* < 0.05; *n* = 31,253 cells from 330 regions of interest (ROIs) from 12 eyes of 8 participants; data from first visit only), reminiscent of the irregularly dilated inner segments observed in a mouse model of *RHO*-RP and a histopathologic study showing swollen cone inner segments^16^. Whereas the cones in healthy mosaics appear to follow a uniform spacing between neighboring cones, the cones in *RHO-*RP appeared to be more irregularly spaced, with clusters of cones observed and increased variability in the cell-to-cell spacing between immediate neighbors. These initial findings highlight subtle but quantifiable changes in cone structure and organization.

**Fig. 3:**
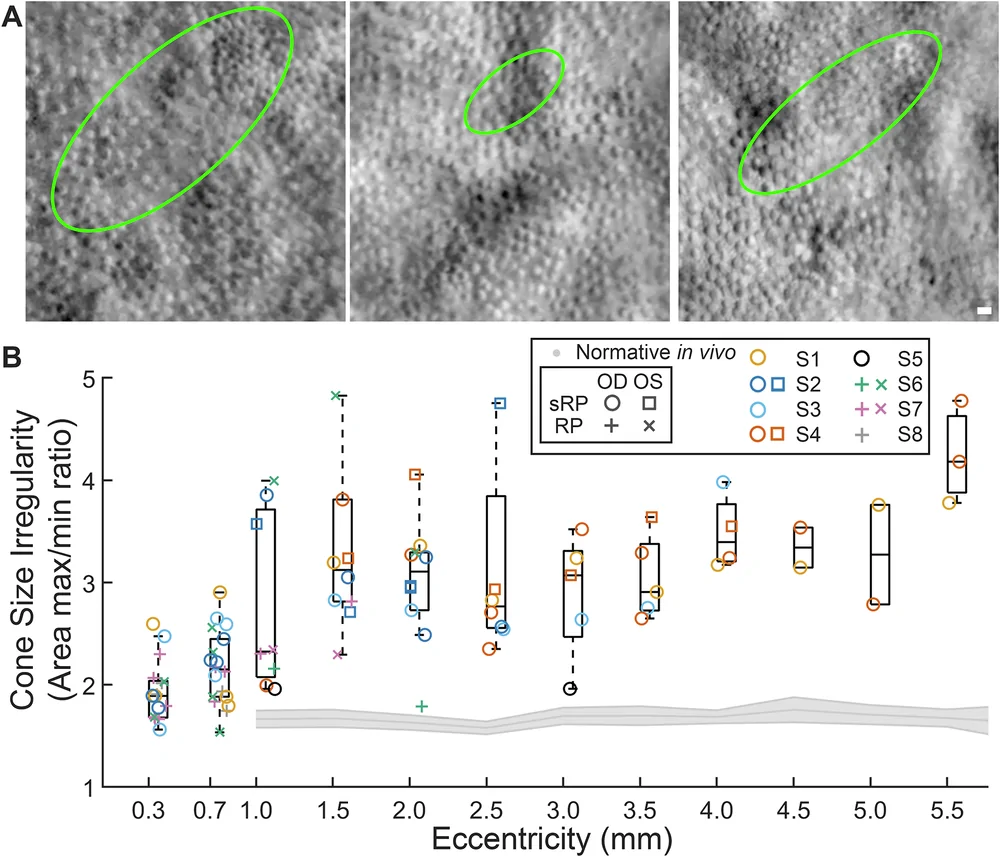
Cones in *RHO-*RP exhibit larger size variability compared to healthy controls. **A** Split detection AO images showing examples of smaller cones interspersed among larger neighboring cones in subject S6R. Green ellipses highlight examples in which the cones are smaller in the upper right and larger in the lower left of each ellipse. Scale bar, 10 µm. **B** Quantification of the ratio between the largest and smallest cone inner segment areas within a single region of interest (ROI) shows a higher degree of variability across all eccentricities in the *RHO-*RP cohort when compared to normative healthy in vivo data (normative data^14,15^ only available for eccentricities 1.0 mm and above; gray shaded region indicates 95% CI around the mean). Each colored symbol within the box plots represents the average of three or more cone ROIs measured at each eccentricity for one eye (see inset legend).

To further explore the increased variability in size between neighboring cones with unusual instances of smaller and larger cones observed to be in close proximity to each other (Fig. 3A), we quantified the ratio between cones with the largest areas divided by cones with the smallest areas (cone size irregularity = max area/min area within an ROI). Cone size irregularity was increased by an average of 96.2 ± 25.4% compared to normal across the range of eccentricities in the *RHO-*RP cohort (*P* < 0.05; *n* = 12 eyes of 8 participants, computed from the same annotations used for cone diameter calculation) (Fig. 3B). Similar to the trend observed for cone diameter, cone size irregularity was smallest closest to the fovea (normative data not available), and larger at eccentricities outside of the parafovea (eccentricity ≥1.0 mm), in support of the model that changes in cone structure are greatest in the presence of rods.

## Rod photoreceptors are affected to a greater extent than surrounding cells near the transition zone

**Fig. 4:**
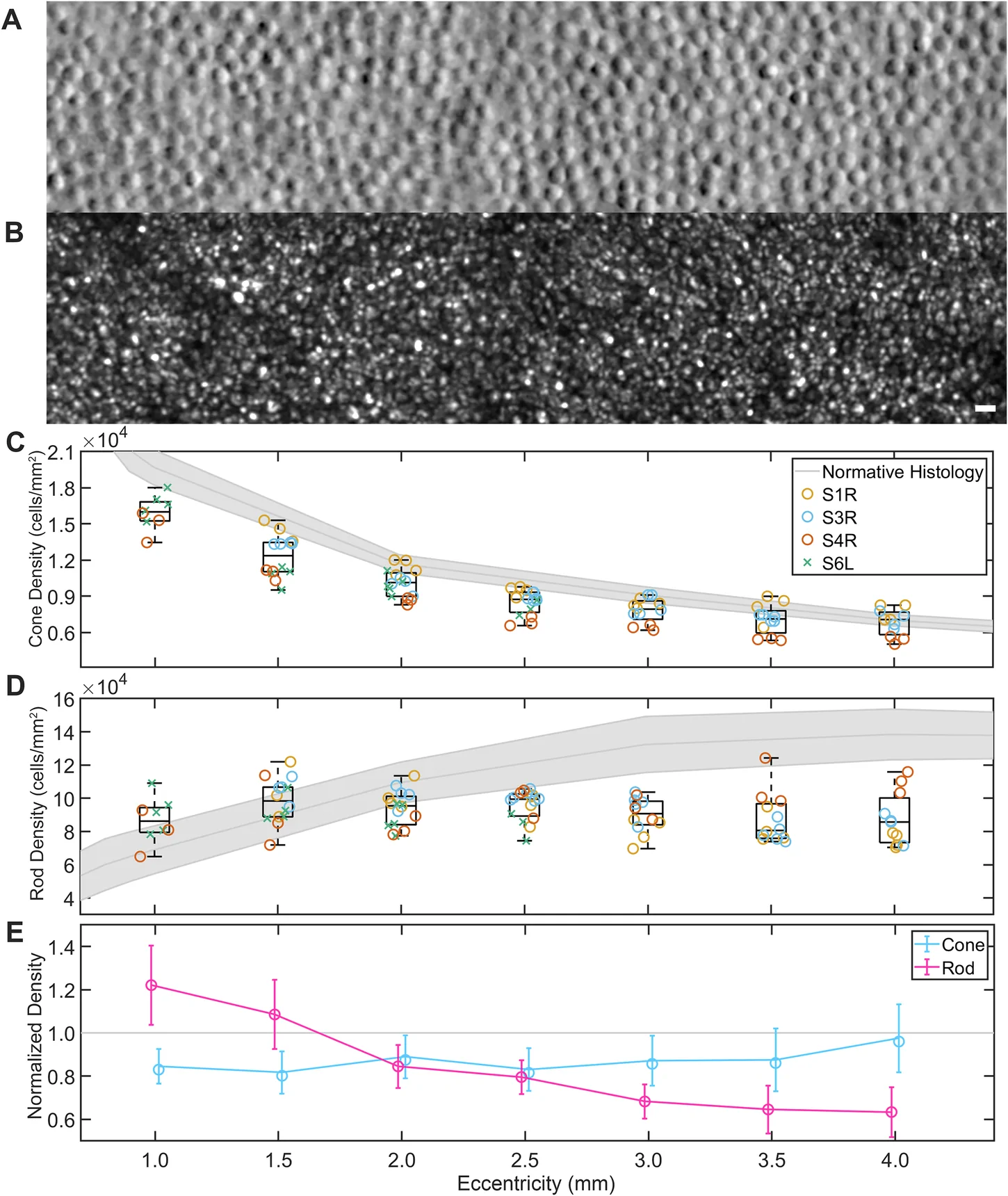
Rod photoreceptor density is decreased to a greater extent than cone density. **A** Split detection AO image showing cones (2.5 mm eccentricity from S3R). **B** Simultaneously-acquired, co-registered confocal reflectance AO image corresponding to the same region as (**A**) showing both cone and rod reflections (cones: central reflection with dark surrounding area; see (**A**) for comparison; rods: single smaller reflection surrounding the cones). Scale bar for both (**A**, **B**), 10 µm. There are small gaps visible in between individual rods, implying reduced rod density. **C**, **D** Quantification of cone and rod density in *RHO*-RP (box plots with individual colored datapoints overlaid) compared to normative histological data^19^ (gray shaded area is the 95% CI around the mean). Each colored datapoint represents a measurement from a single ROI at each eccentricity for one eye (see inset legend). **E** In these eyes, normalized cone and rod density values (*RHO*-RP divided by histology) showing a greater reduction in rod density relative to cone density in comparison to normal (gray horizontal line) at the largest eccentricities and normal or supranormal values at the smallest eccentricities.

Indeed, direct quantification of both rods and cones revealed a greater disruption to rod density than to cone density at the leading disease front (Fig. 4). Although direct quantification of rods is typically challenging in healthy eyes due to their small size (high density) relative to the resolution limit of AO imaging^17,18^, the success rate of imaging rods in *RHO*-RP was unexpectedly high, consistent with the decreased rod density in these eyes; notably, this was not due to shorter axial lengths which would facilitate higher resolution imaging (24.16 ± 0.75 mm across the *RHO*-RP cohort, average ± SD). On average, cone density was reduced by 14.30 ± 5.24% (*P* < 0.05 for most eccentricities; *n* = 5967 cells from 89 ROIs from 4 eyes of 4 participants) (Fig. 4C), consistent with previous reports^10^. In comparison, rod density was significantly reduced at larger eccentricities (*P* < 0.05) but approached normal or even supranormal values at smaller eccentricities (Fig. 4D). Cone density was similar to normative values^19,20^ near the fovea (*P* = 0.18) (Supplementary Fig. 2) (rods were not identified near the fovea, as expected from histological data^19^), consistent with a prior AO study quantifying foveal and parafoveal cones in retinitis pigmentosa^12^. Although there was a relatively uniform reduction in cone density across the retina, the greatest morphological changes (cone size irregularity) were observed in the areas with greatest rod loss (i.e., decrease in rod density). This suggests that changes in cell morphology may be indicative of rod loss and provides a potential opportunity to apply cone morphology-based imaging biomarkers to monitor early rod disease.

Although there were significant reductions in both cone and rod density near the disease front, this did not appear to extend to the underlying retinal pigment epithelium (RPE). Typically, the RPE is obscured by the photoreceptors in healthy eyes. However, RPE can sometimes be directly observed using reflectance imaging after loss of photoreceptors^21–23^. Based on this approach we found an intact RPE layer in subject S2L that did not appear to be disrupted even after loss of the outer retina (Supplementary Fig. 3). Quantification of RPE in this area of photoreceptor loss (to the right of the dashed line in Supplementary Fig. 3) revealed an RPE density of 6219 cells/mm^2^, within expected normative values^24^ (95% confidence interval (CI): 5701 to 6343 cells/mm^2^), suggesting that the rod-driven changes in *RHO-*RP have a direct effect on cone structure, but not on RPE structure.

Our data are in agreement with the view that *RHO-*RP primarily affects the rod photoreceptors at the leading disease front but adds new evidence for changes in the structure and regularity of cone photoreceptor structure at the cellular level. Although changes in cone density and diameter are relatively mild, our investigations across a cohort that includes participants with sRP demonstrate that there are widespread cone changes early in disease in relatively healthy and well-preserved retina. These cellular-scale structural changes, which include larger than expected variation in cone size (Fig. 3), may be the long-term result of rod dysfunction, as confirmed by ffERG (Supplementary Table 1), prior to overt degeneration and photoreceptor loss. In addition to the presence of enlarged cone inner segments, we observed an abnormal clustering of cones (i.e., not regularly spaced) that seemed to be characteristic across the *RHO*-RP cohort, especially in areas near the boundary between preserved and diseased retina. This degree of cone disarray can also be observed in a previous report applying split detection AO imaging to RP^12^.

## Cone photoreceptors were affected to a greater extent in RP compared to sRP

Although similar trends were observed across both sRP and RP, there was a slightly greater severity observed for RP than for sRP, which is generally considered to be milder and more slowly progressing. Restricting the data to only a subset of eccentricities at which both sRP and RP data overlapped, we found that cone enlargement was 24.2 ± 5.6% for RP compared to 16.1 ± 13.8% for sRP (eccentricity range 0.3 to 2.5 mm) (Fig. 2C). Cone size irregularity was increased by 129.4 ± 116.3% above normal for RP compared to 90.0 ± 17.6% for sRP (eccentricity range 1.0 to 2.5 mm; normative data not available below 1.0 mm) (Fig. 3B). Finally, parafoveal cone density for RP was −23.3 ± 13.9% below normal compared to −6.0 ± 8.6% for sRP (Supplemtnary Fig. 2; insufficient data for comparison beyond 1.0 mm eccentricity). Taken together, these findings support the understanding that sRP is a milder phenotype compared to RP but nonetheless highlight similarities in cone disorganization. Our measurements of cellular structure were consistent with functional ffERG measurements, which indicated that cone function more robust than rod function, especially in the participants with sRP compared to those with RP (Supplementary Table 1). Similar to previous papers, we did observe a slow progression in sRP of the NIRAF boundary advancing toward the fovea across the follow up period^13^.

## Longitudinal assessment of cone structure

For a subset of participants who agreed to return for longitudinal follow up (Table 1), we repeated measurements of cone structure to investigate signs of disease progression at the cellular level (*n* = 2708 cones from 30 ROIs; 5 eyes from 5 participants; 25.2 ± 18.2 months between visits 1 and 2; 20.4 ± 10.4 months between visits 2 and 3; mean ± SD). Despite an average total follow up of 3.8 years, normalized measurements^25^ of cone inner segment diameter, cone density, and cone size irregularity were not significantly different across the visits for both the parafovea and transition zone locations (Supplementary Fig. 4A–C). Although there were some changes within specific eyes, overall, normalized cone diameters were consistently larger than normal across visits, corroborating our cross-sectional measurements of cone diameter (Fig. 2). Similarly, overall, cone density was slightly reduced for both locations across visits, corroborating our cross-sectional measurements of cone density (Fig. 4C). These repeated measurements confirm that the changes in cone structure that we observed in *RHO*-RP are stable in relatively well-preserved retinal regions and can be reliably quantified across a period of several years.

Finally, to further capture the observed irregularity in cell-to-cell topography that we qualitatively identified across all participants, we quantified the percentage of cones that did not follow a hexagonal packing arrangement (i.e., 6 neighbors). Overall, cones in the transition zone were much less likely to follow a hexagonal packing arrangement compared to cones in the parafovea; these changes were stable across visits (Supplementary Fig. 4D). These differences illustrate the possibility of disruption to the cone mosaic topography at the rod-driven leading front of disease.

As one of the first genes attributed to RP, there remains a relatively high interest in treatment trials for *RHO*-RP^5,13^. Direct quantification of rod photoreceptors near the transition zone may be useful for monitoring disease progression in sRP. However, given that quantification of rod structure remains one of the most challenging technical capabilities of modern AO systems, characterization of cone photoreceptor disruption and disorganization in retinal locations that do not necessarily need to be restricted to the disease front might provide more accessible markers for upcoming treatment trials aimed at preserving photoreceptor structure. Detection and monitoring of structural photoreceptor abnormalities that precede vision loss can ultimately inform therapeutic timing or potentially serve as structural outcome measures in preparation for upcoming clinical trials.

## Methods

### Clinical evaluation of participants

Participants diagnosed with *RHO*-associated retinitis pigmentosa were recruited from the National Eye Institute eye clinic for this study. Participants who had unstable fixation, media opacity, or poor central visual acuity were excluded. All participants underwent genetic testing from clinical genetic testing laboratories which identified pathogenic variants in *RHO* (Transcript ID: NM_000539.3). Clinical evaluation included best-corrected visual acuity, ocular biometry (IOL Master, Carl Zeiss Meditec), dilated funduscopic examination, fundus photography (Topcon), and spectral domain optical coherence tomography (SDOCT) (Spectralis, Heidelberg Engineering). We also performed near-infrared fundus autofluorescence (NIRAF)^24^ to detect the disease front on the basis of autofluorescence of melanin; NIRAF uses a longer wavelength of light (790 nm) than the 488 nm used for short-wavelength fundus autofluorescence (Spectralis, Heidelberg Engineering). International Society for Clinical Electrophysiology Vision^26^ full-field flash electroretinograms (ffERG) were recorded using bipolar Burian-Allen contact lens electrodes.

Eight participants from six families with *RHO*-RP were included in this study with a mean age of 41.8 years (Table 1). Five participants were described to have sRP based on fundus appearance, were confirmed to have more severe retinal degeneration inferiorly, and had a clear border demarcating the boundary between diseased and preserved retina on NIRAF (Supplementary Fig. 1). The remaining three participants had RP (i.e., not sRP). The fundus appearance and clinical disease were symmetric between eyes in this cohort. Most pathogenic variants could be classified as class 2, characterized by retention of misfolded protein in the endoplasmic reticulum^5,13^. Co-registration of NIRAF and AO images with clinical SDOCT confirmed the presence of intact outer retinal bands corresponding to the area of preserved retina, with the external limiting membrane descending near or slightly beyond the ellipsoid zone band in all eyes (Fig.1).

### Adaptive optics retinal imaging

A total of 12 eyes from 8 participants were imaged using a custom-built multimodal adaptive optics instrument incorporating both confocal reflectance^6,21^ and non-confocal split detection^7^. Average light power levels measured at the corneal plane were maintained below 135 µW for the 790 nm light source (retinal imaging) and below 40 µW for 880 nm (wavefront sensing), below the maximum permissible exposure defined by the American National Standards Institute Z80.36-2021 standard. Before imaging, eyes were dilated with 2.5% phenylephrine hydrochloride and 1% tropicamide. To image cone and rod photoreceptors, approximately 50–150 overlapping locations were imaged per eye, per visit, starting with the central macula and extending out temporally to approximately 5.5 mm (or less if there was no remaining ellipsoid zone band on OCT). The preferred retinal locus of fixation was obtained by asking subjects to look at the center of the dim red window of imaging light as a reference for the foveal center. Subjects were advised to blink naturally. Participants who were willing to return for follow-up visits were invited for follow-up adaptive optics imaging.

### Cone and rod photoreceptor measurements

After correcting the adaptive optics videos for eye motion, overlapping AO images were assembled into montages and registered to clinical images. ROIs were manually selected at fixed eccentricities in the temporal direction, skipping any locations that were beyond the leading disease front, avoiding the presence of large blood vessels, and adjusted in size to allow for approximately 100 cells within a square (10 × 10 cells, height×width). Cone photoreceptors were identified on the basis of their asymmetrical shading (dark on the left and bright on the right)^7,27^ and cone inner segment boundaries were segmented from non-confocal split detection adaptive optics images in a semi-automated fashion, starting with automated algorithms^28,29^, followed by iterative manual adjustments by expert graders until full consensus of all marks was achieved in at least two graders. Similarly, cone and rod photoreceptor cell centers are identified using automated algorithms^28,30^ followed by iterative consensus grading, based on direct counting^17^. Simultaneously acquired confocal reflectance and non-confocal split detection images were compared against each other to facilitate the identification of rods vs. cones (Supplementary Video 1).

Cone diameters were calculated as the diameter of the circle with equivalent area^28,29^. Rod and cone density were calculated as the total number of cells divided by the area of the Voronoi regions fully contained within the ROI, discarding Voronoi regions at the borders of ROIs that extended beyond the ROI boundary. To generate normalized measures of cone and rod metrics^25^, each measured value was divided by the expected normative value for the corresponding retinal eccentricity.

For repeated longitudinal measurements, we considered two locations for longitudinal analysis across three visits: one at a fixed eccentricity of 1.0 mm temporal to the fovea (“parafovea”) and the second at an eccentric temporal location near the boundary between preserved and absent photoreceptors on the third visit (“transition zone”). For the transition zone ROI selection, which was performed on the third visit, the same retinal location was propagated back to the first two visits. Visits were co-registered using retinal vascular landmarks to ensure that the same ROIs were consistently selected across visits (Supplementary Fig. 5).

The retinal magnification factor to scale images from degrees to millimeters was computed using a paraxial ray trace after updating a three-surfaced simplified model eye with ocular biometry measurements from each eye, obtained after dilation (axial length, corneal curvature, and anterior chamber depth)^30^.

### Statistical analysis

Statistical analysis was performed to compare *RHO-*RP data to normative data. A two-sided paired t-test was used to compare cone diameters and cone size irregularity in *RHO*-RP to normative data. 95% CIs were calculated for normative cone and rod density for comparison to *RHO*-RP. A one-way analysis of variance (ANOVA) test was used to compare longitudinally acquired data across visits. For all tests, a significance value of 0.05 was used.

### Study approval

Research procedures adhered to the tenets of the Declaration of Helsinki. Written, informed consent was obtained from all participants after the nature of the research and possible consequences of the study were explained. This study was approved by the Institutional Review Board of the National Institutes of Health (15-EI-0020). Although not a clinical trial, this study is registered on clinicaltrials.gov (NCT02317328; registration date 2014-12-13).

## Supplementary information

## Data Availability

All data needed to evaluate the conclusions in the paper are present in the manuscript and/or supporting information.
