# Supplementary material for: In vivo adaptive optics retinal imaging reveals rod-mediated cone photoreceptor disorganization in RHO-associated retinitis pigmentosa
Source: NPJ Biomed Innov. 2026 Sep 2;3:53. doi: 10.1038/s44385-026-00108-3 (PMC13538484; doi:10.1038/s44385-026-00108-3)
Supplement: Supplementary file 1 [file 44385_2026_108_MOESM1_ESM.pdf]

Supplementary Figures

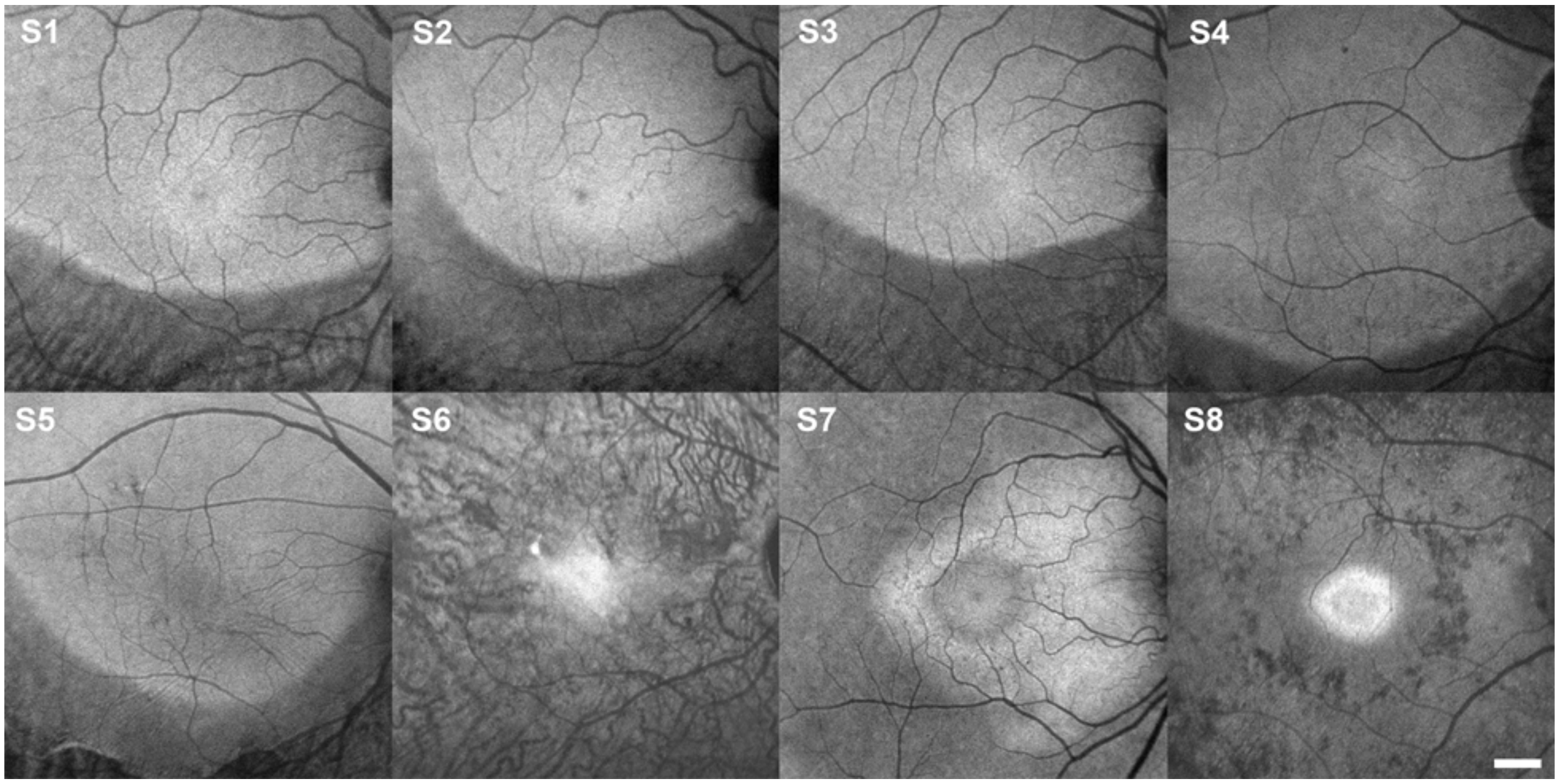

Supplementary Figure 1. Overview of *RHO*-associated retinitis pigmentosa (*RHO*-RP) cohort. Near-infrared autofluorescence (NIRAF) images (top left indicates ID in **Table 1**). Five participants with sectoral retinitis pigmentosa (sRP) had more severe retinal degeneration inferiorly (S1-S5) and three participants with generalized retinitis pigmentosa (hereafter referred to as RP) showed a more circular autofluorescence pattern (S6-S8). Scale bar, 1 mm.

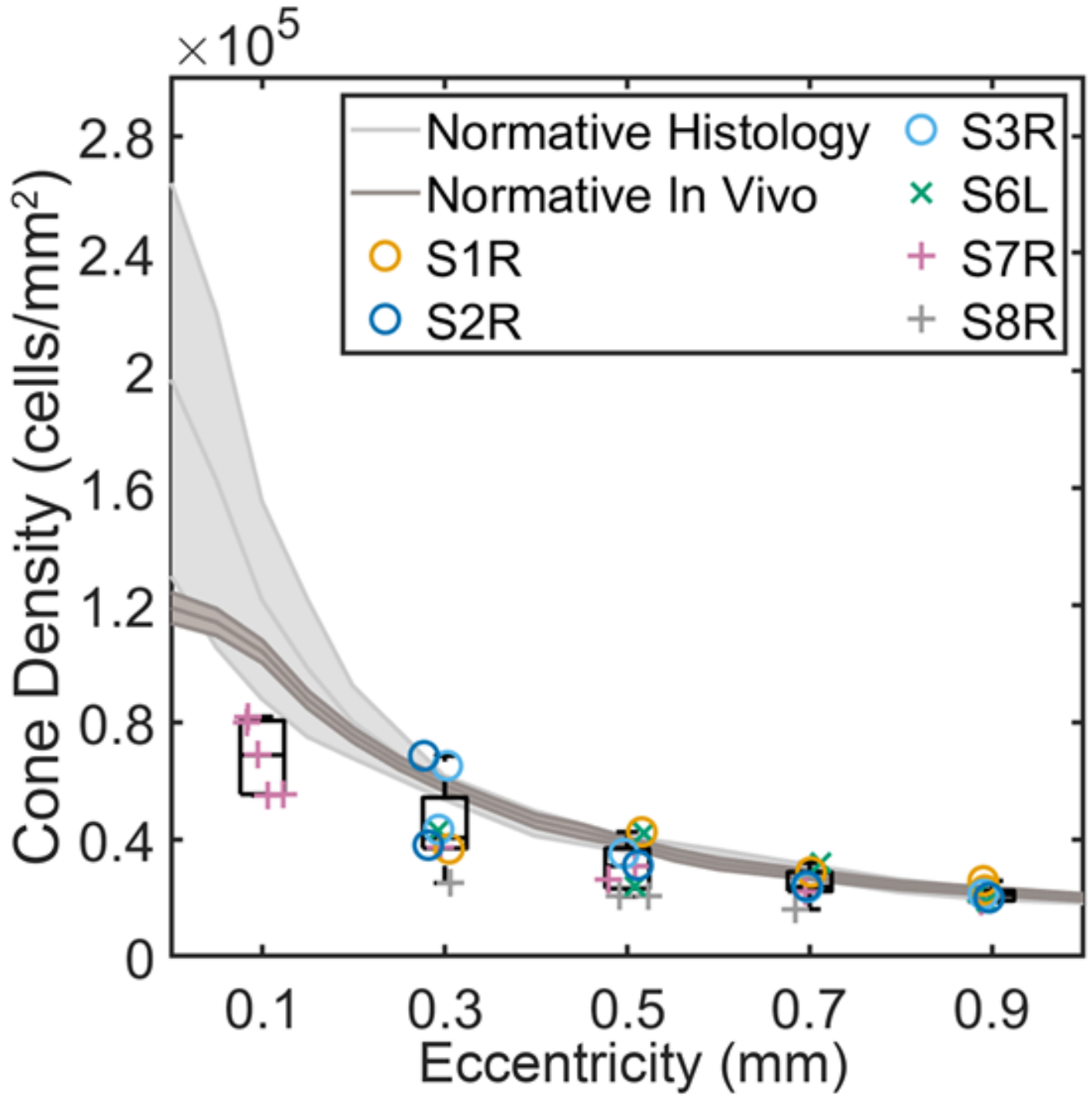

Supplementary Figure 2. Parafoveal cone density in *RHO*-RP. Box plots with datapoints representing measurements obtained from a single ROI are overlaid. Cone density is slightly reduced near the fovea but approaches normative values further from the fovea. Gray shaded area represents 95% confidence intervals (CI) around the mean for normative data (normative histological data[1] in lighter gray and normative in vivo data[2] in darker gray). Participant S7 also had increased cone inner segment diameter near to fovea (**Fig.2**).

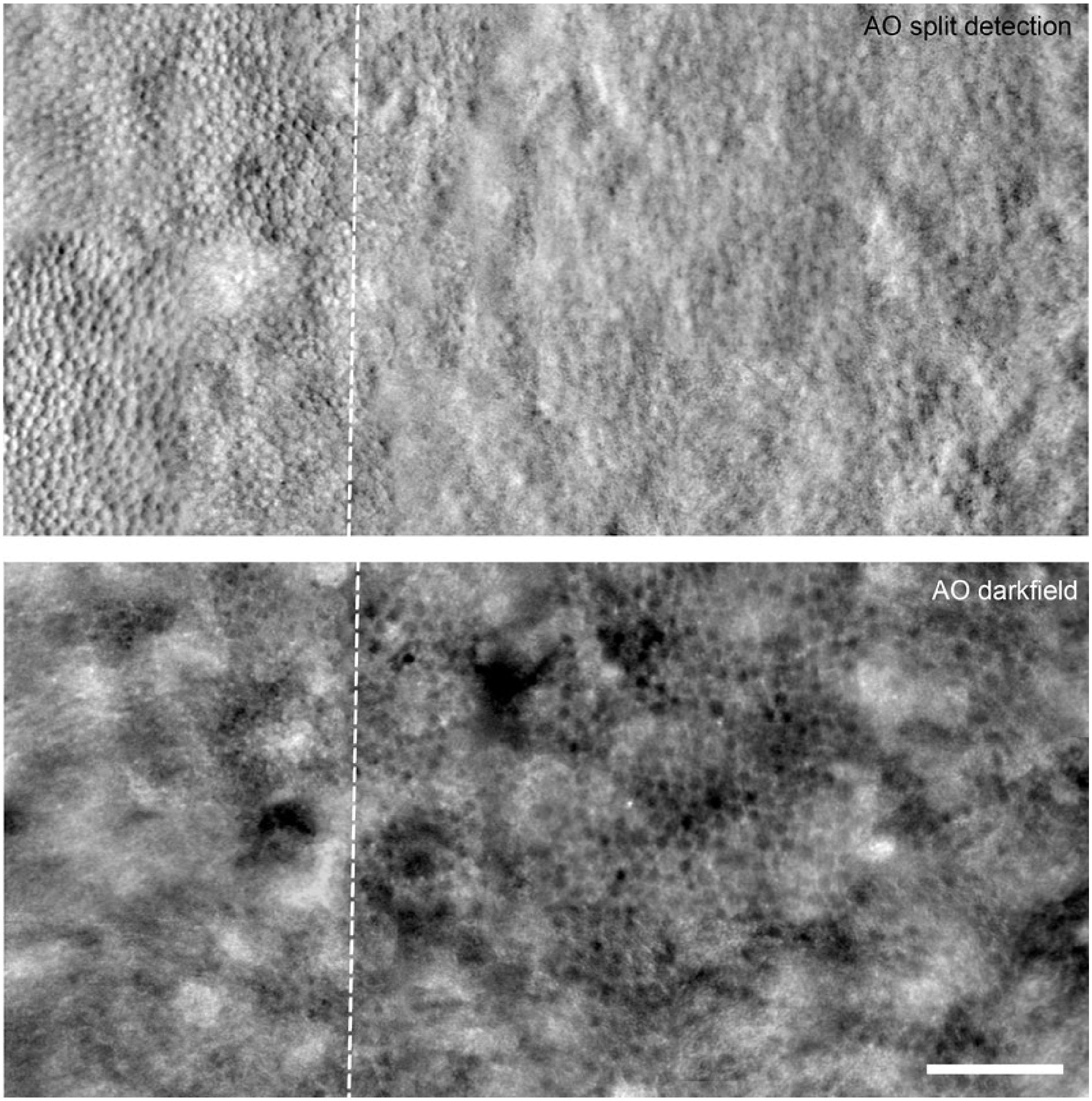

Supplementary Figure 3. AO images at the boundary between diseased (right of dashed line) and preserved (left of dashed line) retina in S2L. Intact cone photoreceptors are visible within the preserved region. An intact mosaic of retinal pigment epithelial (RPE) cells can be seen to the right of the dashed line due to the absence of overlying cone photoreceptors. In the diseased area (right of dashed line), the ellipsoid zone was absent on SDOCT. Scale bar, 100 µm.

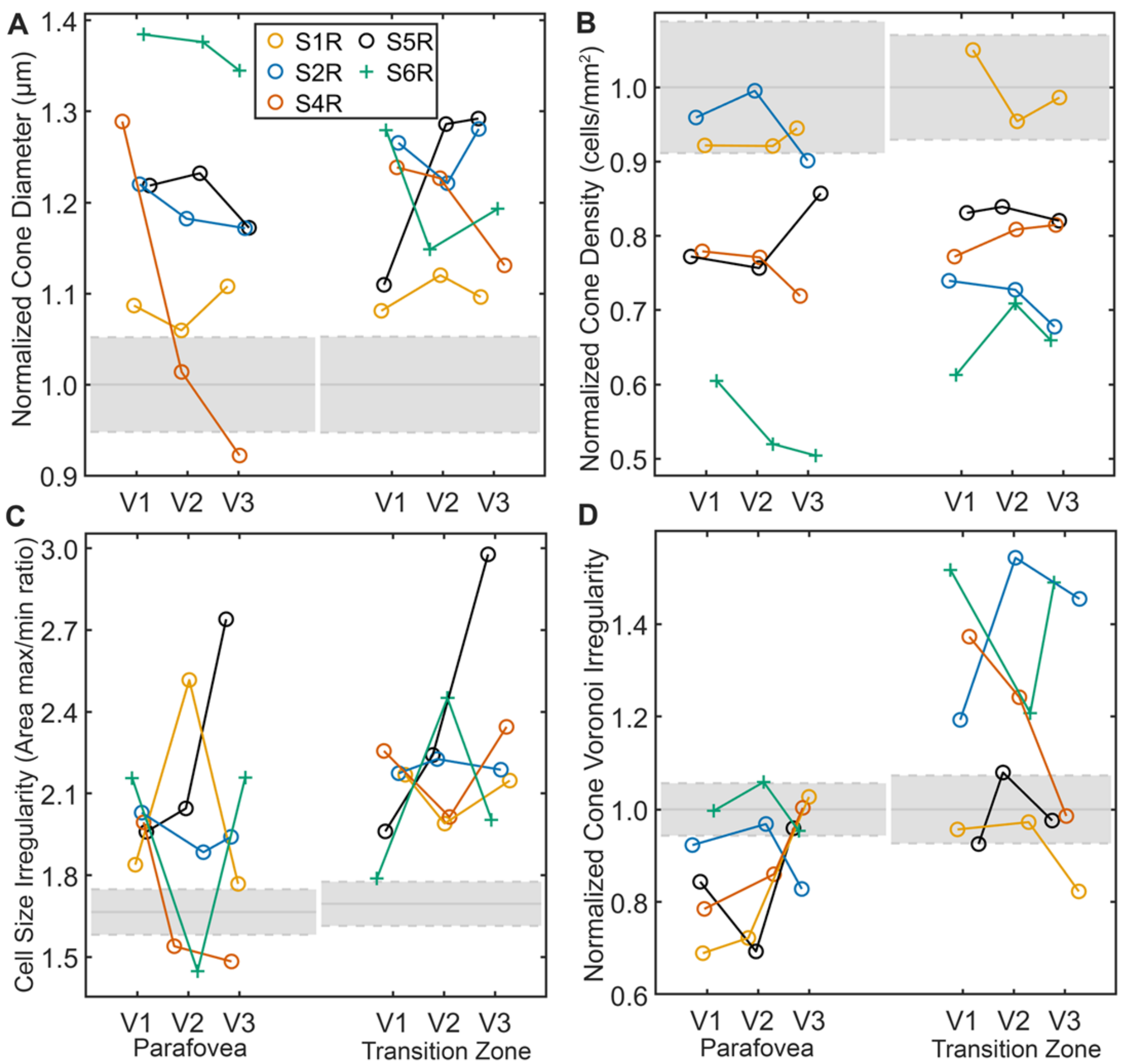

Supplementary Figure 4. Longitudinal assessment of cone structure across three visits (V1, V2, V3). Measurements were obtained in two regions: one at a fixed eccentricity of 1.0 mm (parafovea) and the second at a fixed eccentricity temporal to the fovea in an area of preserved retina immediately adjacent to the NIRAF border of visit 3 (transition zone). **(A)** Normalized[3] cone inner segment diameter measurements were consistently enlarged across visits and were similar across visits in the parafovea (P=0.54) and transition zone (P=0.99). **(B)** Normalized[1] cone density measurements were consistently below normal across visits and were also similar in the parafovea (P=0.98) and transition zone (P=0.98). **(C)** Cell size irregularity was consistently larger than normal[3] across visits and were slightly higher in the transition zone (P=0.34) compared to the parafovea (P=0.84). **(D)** Normalized[2] cone Voronoi irregularity (percentage of cones without six neighbors relative to expected normal values) was larger than expected only in the transition zone but were stable across visits (parafovea, P=0.35; transition zone, P=0.92). Each colored datapoint represents a measurement from a single ROI from a single visit (registered and matched across all three visits). Expected normative values corresponding to these retinal eccentricities (95% CI around the mean) are shown in gray.

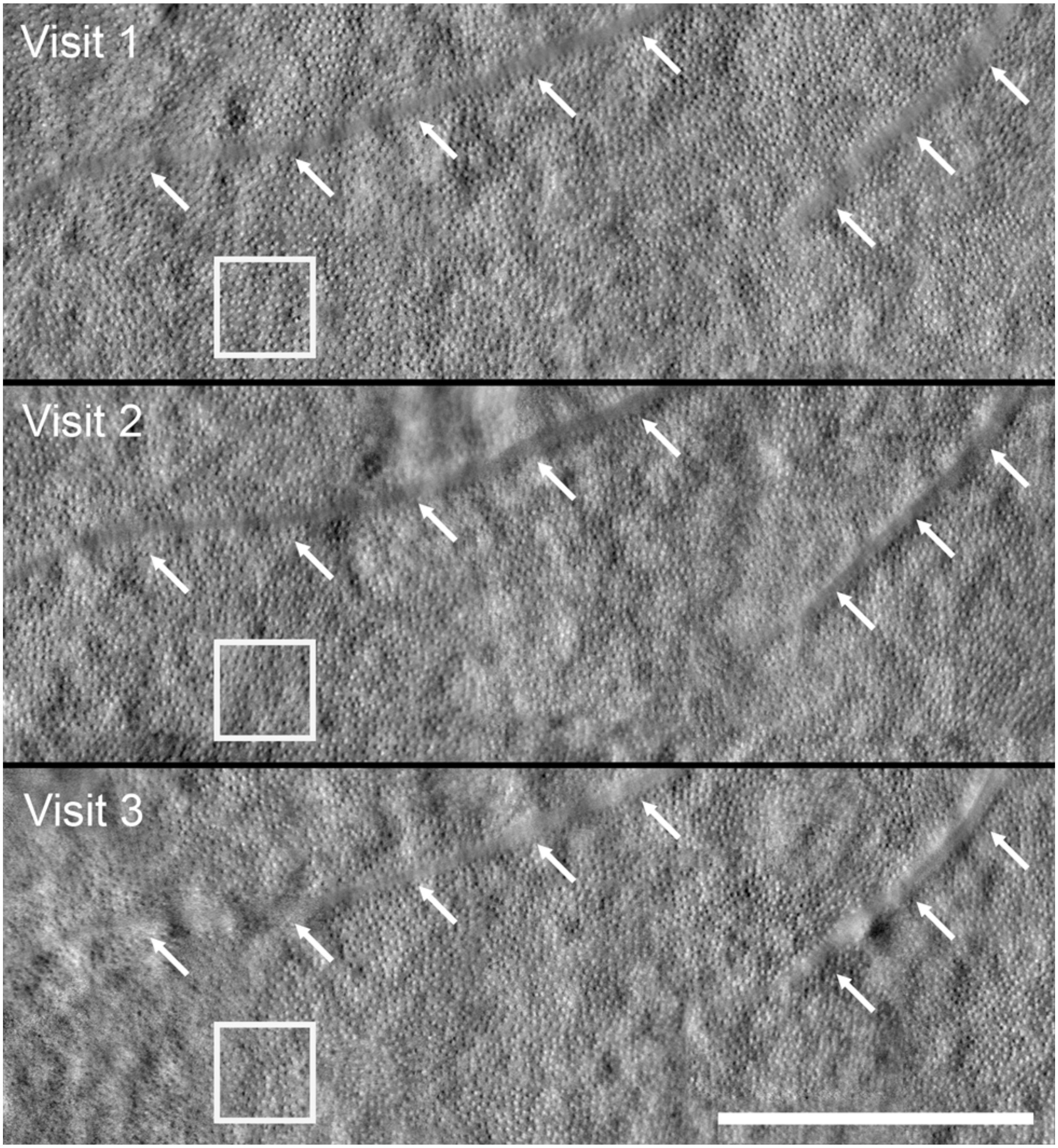

Supplementary Figure 5. Multi-visit registration of AO regions using fiduciary vascular landmarks for subject S4R. Arrows denote retinal vessels that can be seen across all visits. The white box indicates the ROI that was selected as the transition zone ROI in **Supplementary Figure 4**. The cone inner segments are not as clearly visible on the far left edge of the visit 3 image due to the proximity of the ROI to the edge of the transition zone. The transition zone edge is closest to the ROI in visit 3 and furthers from the ROI in visit 1. Scale bar, 0.5 mm.

Supplementary Tables

Supplementary Table 1. Summary of full field electroretinogram (ffERG) measurements

Subjects	DA0.01	LA3	30 Hz
All	68±71	65±35	52±24
sRP	99±72	77±33	61±23
sRP*	75±57	65±26	56±22
RP	18±29	46±31	36±19
Normal	>167	>101	>66

All values are reported as mean±SD, in µV. The sRP group includes subjects S1 to S5 and the RP group includes subjects S6 to S8. The sRP* cohort removes subject S3, who was younger than the other participants with sRP, from the calculation; this subject had normal DA0.01, LA3, and 30Hz measurements recorded in both eyes.

Supplementary Videos

Supplementary Video 1. Co-registered non-confocal split detection and confocal reflectance images of cone and rod photoreceptors corresponding to subject S3R shown in **Fig.4AB**. Cone inner segments are more readily visible in the non-confocal split detection channel (dark on the left, bright on the right of each cone inner segment). Both cones and rods are visible in the confocal reflectance channel (larger dots surrounded by a dark ring correspond to cones and are colocalized with cone inner segments in visible in the simultaneously-acquired non-confocal channel; smaller dots which are not colocalized with cone inner segments correspond to rods).

Supplementary References

1. Curcio CA, Sloan KR, Kalina RE, Hendrickson AE. Human photoreceptor topography. J Comp Neurol 1990;292:497–523.

2. Cooper RF, Wilk MA, Tarima S, Carroll J. Evaluating Descriptive Metrics of the Human Cone Mosaic. Investig Opthalmology Vis Sci 2016;57:2992.

3. Aguilera N, Iyer S, Cruz MJ, et al. Normative adaptive optics retinal imaging reveals age and sex dependent differences in cone photoreceptor diameter. Invest Ophthalmol Vis Sci 2024;65:1421–1421.
